# Cost-effectiveness of a fixed combination of netupitant and palonosetron (NEPA) relative to aprepitant plus granisetron (APR + GRAN) for prophylaxis of chemotherapy-induced nausea and vomiting (CINV): a trial-based analysis

**DOI:** 10.1007/s00520-019-04824-y

**Published:** 2019-06-03

**Authors:** Marc Botteman, Katharina Nickel, Shelby Corman, Marco Turini, Gary Binder

**Affiliations:** 1 Pharmerit International, 4350 East West Highway, Suite 1100, Bethesda, MD 20814 USA; 2 Pharmerit International, Berlin, Germany; 3 Helsinn Healthcare SA, Pazzallo, Lugano, Switzerland; 4 Helsinn Therapeutics US, Inc., Iselin, NJ USA

**Keywords:** Antiemetic, Cost-effectiveness, Netupitant and palonosetron, Chemotherapy, Nausea, CINV

## Abstract

**Purpose:**

To assess, from a United States (US) perspective, the cost-effectiveness of chemotherapy-induced nausea and vomiting (CINV) prophylaxis using a single dose of netupitant and palonosetron in a fixed combination (NEPA) versus aprepitant plus granisetron (APR + GRAN), each in combination with dexamethasone, in chemotherapy-naïve patients receiving highly emetogenic chemotherapy (HEC).

**Methods:**

We analyzed patient-level outcomes over a 5-day post-HEC period from a randomized, double-blind, phase 3 clinical trial of NEPA (*n* = 412) versus APR + GRAN (*n* = 416). Costs and CINV-related utilities were assigned to each subject using published sources. Parameter uncertainty was addressed via multivariate probabilistic sensitivity analyses (PSA).

**Results:**

Compared to APR + GRAN, NEPA resulted in a gain of 0.09 quality-adjusted life-days (QALDs) (4.04 vs 3.95; 95% CI −0.06 to 0.25) and a significant total per-patient cost reduction of $309 ($943 vs $1252; 95% CI $4–$626), due principally to $258 in lower medical costs of CINV-related events ($409 vs $668; 95% CI −$46 to $572) and $45 in lower study drug costs ($531 vs $577). In the PSA, NEPA resulted in lower costs and higher QALD in 86.5% of cases and cost ≤ $25,000 per quality-adjusted life-year gained in 97.8% of cases.

**Conclusions:**

This first-ever economic analysis using patient-level data from a phase 3 trial comparing neurokinin-1 receptor antagonist (NK1 RA) antiemetic regimens suggests that NEPA is highly cost-effective (and in fact cost-saving) versus an aprepitant-based regimen in post-HEC CINV prevention. Actual savings may be higher, as we focused only on the first chemotherapy cycle and omitted the impact of CINV-related chemotherapy discontinuation.

**Electronic supplementary material:**

The online version of this article (10.1007/s00520-019-04824-y) contains supplementary material, which is available to authorized users.

## Introduction

Without adequate antiemetic prophylaxis, as many as 90% of patients receiving highly emetogenic chemotherapies (HEC) experience chemotherapy-induced nausea and vomiting (CINV) [1]. CINV is among the most distressing and feared side effect cancer patients experience during chemotherapy and is associated with significant quality-of-life impairment [2]. Moreover, CINV may cause a delay or discontinuation of therapy, and studies indicate overall survival benefits in patients whose chemotherapy symptoms have been monitored systematically between visits [3]. Uncontrolled CINV can lead to a substantial financial burden due to unscheduled physician and emergency department (ED) visits and hospitalizations [4, 5]. The importance of CINV prevention is further recognized by the inclusion of nausea and vomiting among the causes of avoidable ED visits and hospitalizations within the Centers for Medicare & Medicaid Services (CMS) outcomes-based quality measure for outpatient cancer care (OP-35) [6].

Various guidelines recommend specific prophylaxis regimens at initiation of each cycle of HEC to prevent CINV. The most commonly used prophylaxis for HEC is a 3-drug combination including a serotonin-3 receptor antagonist (5-HT3 RA), a neurokinin-1 receptor antagonist (NK1 RA), and dexamethasone [7, 8]. Appropriate antiemetic prophylaxis can prevent an estimated 70% to 80% of CINV episodes [9]. Effective prophylaxis during the first cycle of chemotherapy is of particular importance since patients who experience CINV in the initial cycle face higher risk during subsequent ones [10].

Only two injectable NK1 RA agents are available in the United States (US): fosaprepitant/aprepitant (approved by the US Food and Drug Administration [FDA] for injection in 2010 and 2017, respectively [11, 12]) and netupitant/fosnetupitant (FDA-approved for injection in 2018 as a fixed combination with the 5-HT3 RA palonosetron [NEPA] [13]). Each injectable NK1 RA agent was approved based on bioequivalence to the oral formulation and, for NEPA, based on a prospective safety trial [13]. In phase 3 clinical trials, the percentages of patients achieving complete response (CR), defined as no emesis or rescue medication use, were significantly higher for those receiving oral NEPA versus oral palonosetron (PALO) alone during a single cycle of HEC (89.6% vs 76.5%; *P* < 0.050) [14] and over up to 4 cycles (63.6% vs 50.6%; *P* < 0.0001) [15]. Most recently, a phase 3 non-inferiority trial conducted in China, Taiwan, Korea, and Thailand randomized 834 chemotherapy-naïve patients receiving cisplatin-based HEC to a single oral dose of NEPA (*n* = 417) or a 3-day oral aprepitant (APR) plus 1-day intravenous granisetron (GRAN) regimen (*n* = 417), each in combination with oral dexamethasone on days 1 through 4 [16]. The full analysis set (FAS) population (NEPA: *n* = 412; APR + GRAN: *n* = 416) included patients receiving the study prophylaxis as well as HEC treatment [16]. Compared to APR + GRAN, NEPA demonstrated non-inferiority, with favorable overall CR (73.8% vs 72.4%), overall no emesis (75.0% vs 74.0%), no rescue medication (96.6% vs 93.5%), and no significant nausea (75.7% vs 70.4%), with a similar safety profile [16].

While clinical trials and a recent network meta-analysis comparing the efficacy of different triple antiemetic regimens [17, 18] indicate favorable outcomes for NEPA, less is known about the relative cost-effectiveness of NK1 RA-containing regimens in the US. Therefore, this study was conducted to evaluate the US cost-effectiveness of NEPA versus APR + GRAN in CINV prevention based on the patient-level data of the aforementioned phase 3 study [16].

## Methods

### Overview

This economic analysis comparing NEPA to APR + GRAN in the prevention of CINV post-HEC administration was conducted from a US healthcare perspective and included direct CINV prophylaxis and treatment costs as well as quality-adjusted life-days (QALDs) over a single chemotherapy cycle. Efficacy inputs were based on individual patient-level data from the phase 3 non-inferiority clinical trial described above [16]. Healthcare resource use (HCRU), costs of CINV-related events, drug costs, and CINV-related utilities were not collected in the trial and were obtained from the literature and assigned to each trial subject based on their individually observed outcomes (i.e., CINV events) and drug utilization pattern (i.e., antiemetic prophylaxis and rescue medications).

### Efficacy

The efficacy of NEPA and APR + GRAN was derived based on the trial patient-level data [16]. In brief, chemotherapy-naïve patients scheduled to receive their first course of cisplatin-based chemotherapy for a solid tumor were randomized to receive a single oral dose of NEPA or 3 days of oral APR plus a single dose of intravenous GRAN, each in combination with oral dexamethasone on days 1 through 4. Emetic events and rescue medication use were recorded on days 1 through 5. The primary clinical endpoint was CR over the 5-day period post-HEC; the study was powered to assess this endpoint on a non-inferiority basis. The severity of nausea was measured on days 1 through 5 using a 100-mm visual analogue scale (VAS), with 0 mm representing “no nausea” and 100 mm representing “nausea as bad as it could be.” Prespecified secondary endpoints for efficacy included daily CR rates and no significant nausea (i.e., VAS < 25 mm) [16].

The efficacy of CINV prophylaxis on each day (1 through 5) was categorized as complete protection (CP, defined as absence of any of an emetic episode or use of rescue medication and no significant nausea), CR (defined as no emetic episodes or rescue medication use, regardless of VAS score), or incomplete response (IR, all other patients). Efficacy outcomes used in the cost-effectiveness analysis included the proportions of patients with overall CR, severe nausea (VAS > 80 mm), or prolonged CINV (≥ 3 days of emetic episodes or rescue medication use).

Although 417 patients per group were randomized (intent-to-treat [ITT] population), 6 did not receive study drug (NEPA: *n* = 4; APR + GRAN: *n* = 1) or HEC (NEPA: *n* = 1). Thus, the FAS population, which was used in the base-case analysis, included 412 and 416 patients randomized to NEPA and APR + GRAN, respectively. The mean patient age was 54.5 years; most patients were male (71%), 100% were Asian, 98% had an Eastern Cooperative Oncology Group (ECOG) performance status score of 0 or 1, and 66% had lung cancer (Table 1) [16].

**Table 1:** Baseline patient characteristics of the full analysis set (FAS) population

Characteristics	NEPA (*N* = 412)	APR + GRAN (*N* = 416)	Total (*N* = 828)
Age, mean (SD), years	54.5 (9.59)	54.5 (10.24)	54.5 (9.91)
Sex			
Male	291 (70.6%)	297 (71.4%)	588 (71.0%)
Female	121 (29.4%)	119 (28.6%)	240 (29.0%)
Asian ethnicity	412 (100.0%)	416 (100.0%)	828 (100.0%)
ECOG PS score			
0	175 (42.5%)	171 (41.1%)	346 (41.8%)
1	230 (55.8%)	236 (56.7%)	466 (56.3%)
2	7 (1.7%)	9 (2.2%)	16 (1.9%)
Cancer type			
Lung cancer	275 (66.7%)	267 (64.2%)	542 (65.5%)
Not lung cancer	137 (33.3%)	149 (35.8%)	286 (34.5%)
Metastatic disease status			
Yes	176 (42.7%)	136 (32.7%)	312 (37.7%)
No	236 (57.3%)	280 (67.3%)	516 (62.3%)

*APR*, aprepitant; *ECOG*, Eastern Cooperative Oncology Group; *GRAN*, granisetron; *NEPA*, netupitant and palonosetron; *PS*, performance status

There was a numerical advantage in overall CR for NEPA (+1.4 percentage points), but this was not statistically significant (the trial was powered for non-inferiority). On the prespecified secondary endpoint of daily CR, event rates for the two arms were similar for the initial 2 days and then favored NEPA over APR + GRAN, with the difference becoming statistically significant by day 5 (8% vs 13.9%; *P* = 0.0063). There were fewer patients with prolonged CINV in the NEPA arm versus the APR + GRAN arm (8.5% vs 12.3%) [16, 19].

### Utilities

Utilities of 0.90, 0.70, and 0.24 were assigned for the outcomes of CP, CR, and IR, respectively, consistent with previously published economic models for CINV [20, 21]. Quality-adjusted life-days (QALDs) were calculated by summing the patient’s quality-adjusted time over the 5-day trial; given the average age and disease burden, the maximum possible QALDs (i.e., having no CINV) for the period was 4.5 (i.e., 0.90 × 5 days).

### Resource use and cost

Per-patient CINV-related costs included antiemetic prophylaxis, rescue medications, and medical costs of CINV-related events. The costs of antiemetic prophylaxis and rescue medication drug use were assigned to each arm based on usage actually observed in the trial. The January 2018 Medicare average sales price (ASP) [22] plus 6% was used for intravenous products, and January 2018 wholesale acquisition costs (WAC) [23] plus 3% were used for oral products and 1 intravenous rescue medication (esomeprazole) without an available ASP (Table 2 and Supplementary Table 1). Administration costs for injectable medications and a dispensing fee for oral drugs were added based on literature [24, 25] (see Table 2 for details). A $50 patient copayment was assumed for oral study drugs APR and NEPA (Table 2).

**Table 2:** Costs of study drugs and other items

Item	Dosage	Cost^a^	Source
Study drug costs			
Netupitant and palonosetron (oral)	1 capsule	$560.00	Red Book 2018 [23]
Aprepitant (oral)	Pack of 3	$576.99	Red Book 2018 [23]
Granisetron (IV)	3 mg	$9.51	CMS 2018a [22]
Dexamethasone (oral)	7 × 4 mg	$0.10	Red Book 2018 [23]
Other cost items			
Patient copay (oral antiemetic study drug only)		$50.00	Assumption
Dispensing fee (oral products only)		$1.87	PBMI 2015 [25]
Administration cost (IV, SubQ products only)		$18.36	CMS 2018b [24]
Administration cost (intramuscular)		$20.88	CMS 2018b [24]

*CMS*, Centers for Medicare & Medicaid Services; *IV*, intravenous; *SubQ*, subcutaneous; *PBMI*, Pharmacy Benefit Management Institute

^a^Costs in 2018 US dollars

Since HCRU was not recorded in the trial, medical cost per episode of CINV was assigned to patients based on literature values. Specifically, Burke et al. found that 6.4% of patients receiving antiemetic prophylaxis had a CINV-related visit (inpatient, ED, or outpatient hospital) within 6 days post-HEC [5]. While Burke truncated costs for this 6.4% of patients at 6 days post-HEC initiation, CINV-related costs may continue to accrue beyond this initial period. To avoid artificially truncating costs at the 6-day mark, the mean CINV cumulative costs up to 10 days post-HEC reported by Burke et al. was used—i.e., $9920 (derived by multiplying the full-population mean CINV-related 10 days post-HEC cost [$417] across all patients [*n* = 3069], and then dividing by the number of patients who actually incurred CINV related-costs [*n* = 196] within 6 days post-HEC, and adjusting to 2018 US dollars using the medical care component of the consumer price index) [5, 26]. This cost is consistent with findings of other economic assessments of CINV cost [4, 27]. In sensitivity analyses, the effect of truncating HCRU cost at 6 days post-HEC and 50% of the 6-day truncated HCRU cost (i.e., $2593) was investigated.

Assuming patients with the worst nausea VAS scores were most likely to receive medical treatment for CINV, patients in the APR + GRAN study arm were ranked by highest VAS score over the 5 days; the score for the top 6.4% of patients was determined to be greater than 80 mm. To mirror the CINV resource use frequency findings from Burke et al., any patient (regardless of treatment arm) with a VAS greater than 80 mm was assigned a $9920 CINV cost. An alternative approach to assigning patients for resource use was considered, whereby patients in the APR + GRAN study arm were ranked by maximum patient-reported duration of vomiting and/or retching over the 5 days; the duration for the top 6.4% of patients was more than 8.5 h. In this alternative, any patient with a vomiting and/or retching duration more than 8.5 h was assigned the $9920 cost. Separate analyses evaluated different VAS thresholds (± 10 mm from baseline of 80 mm).

### Missing data and imputation

A separate scenario analysis was performed based on the ITT population. Specifically, for NEPA patients who did not receive study drug, drug cost was $0 and the worst CINV-related outcome was assumed (i.e., a cost of $9920 for severe nausea and a utility of IR). Study drug but not CINV costs were assigned to the NEPA patient who received study drug but did not receive HEC. A utility of zero and total cost of $0 were assigned to the APR + GRAN patient who died prior to study drug administration.

### Subgroup analyses

Cost-effectiveness was assessed in subgroups by disease extent (metastatic vs non-metastatic) and cancer type (lung cancer vs other).

### Primary endpoint and analysis for cost-effectiveness

The primary outcome measures of this cost-effectiveness analysis were the net monetary benefit (NMB) and the probability that NEPA is cost-effective versus APR + GRAN at a willingness-to-pay (WTP) per quality-adjusted life-year (QALY) gained threshold of $25,000 or lower ($100,000 in sensitivity analysis) [28]. The NMB was calculated as the QALY gained multiplied by the WTP per QALY ($25,000), to which the cost savings associated with NEPA (relative to APR + GRAN) were added. The NMB is effectively the sum of the monetized QALY gained plus the cost savings. A positive NMB implies NEPA is cost-effective at the $25,000 per QALY threshold; the higher the NMB, the more cost-effective NEPA is. The probability that NEPA is cost-effective at WTP per QALYs gained was derived via multivariate sensitivity analysis combining bootstrapping (for efficacy assumptions) and probabilistic sensitivity analysis (PSA, for all other inputs, i.e., cost and utilities) [29] in 10,000 model simulations. In the PSA, for each of the 10,000 simulations, parameters were drawn from a probability distribution varying rescue medication prices (± 10%), the cost of CINV-related HCRU, and utility values associated with each health state (Table 3). Simultaneously to the PSA, the bootstrap simulated a new trial for each of the 10,000 simulations by drawing (with replacement) from the original trial. Each PSA result is linked to a bootstrapped trial and, as such, uncertainty around the efficacy and other input parameters can be analyzed using simple descriptive statistics such as nonparametric bootstrapped confidence intervals (CIs). The uncertainty of cost estimates and QALDs was estimated by the 2.5 and 97.5 percentile (i.e., bootstrapped CI) [30]. Additional univariate sensitivity analyses covered the scenarios for a ± 25% change in study drug cost difference and a ± 25% change in CINV HCRU cost. All calculations were performed using Microsoft Excel 2016.

**Table 3:** Summary of key model input parameters and distribution for the sensitivity analyses

Item	Mean	SE	Distribution for PSA	Source
Utilities				
Complete protection^b^	0.9	0.18	Beta	Cawston 2017 [20]
Complete response^c^	0.7	0.14	Beta	Cawston 2017 [20]
Incomplete response^d^	0.24	0.048	Beta	Cawston 2017 [20]
HCRU cost^a^				
Cost per patient with severe nausea^e^	$9920	$820	Gamma	Burke 2011 [5]

*HCRU*, healthcare resource use; *PSA*, probabilistic sensitivity analysis; *SE*, standard error

^a^Adjusted to 2018 US dollars

^b^Complete protection (CP) defined as absence of any of an emetic episode or use of rescue medication and no significant nausea (visual analogue scale [VAS] score < 25 mm)

^c^Complete response (CR) defined as no emetic episodes or rescue medication with a VAS score ≥ 25 mm

^d^Incomplete response (IR) if there was no CP or CR

^e^Severe nausea defined as VAS > 80 mm

## Results

**Fig. 1:**
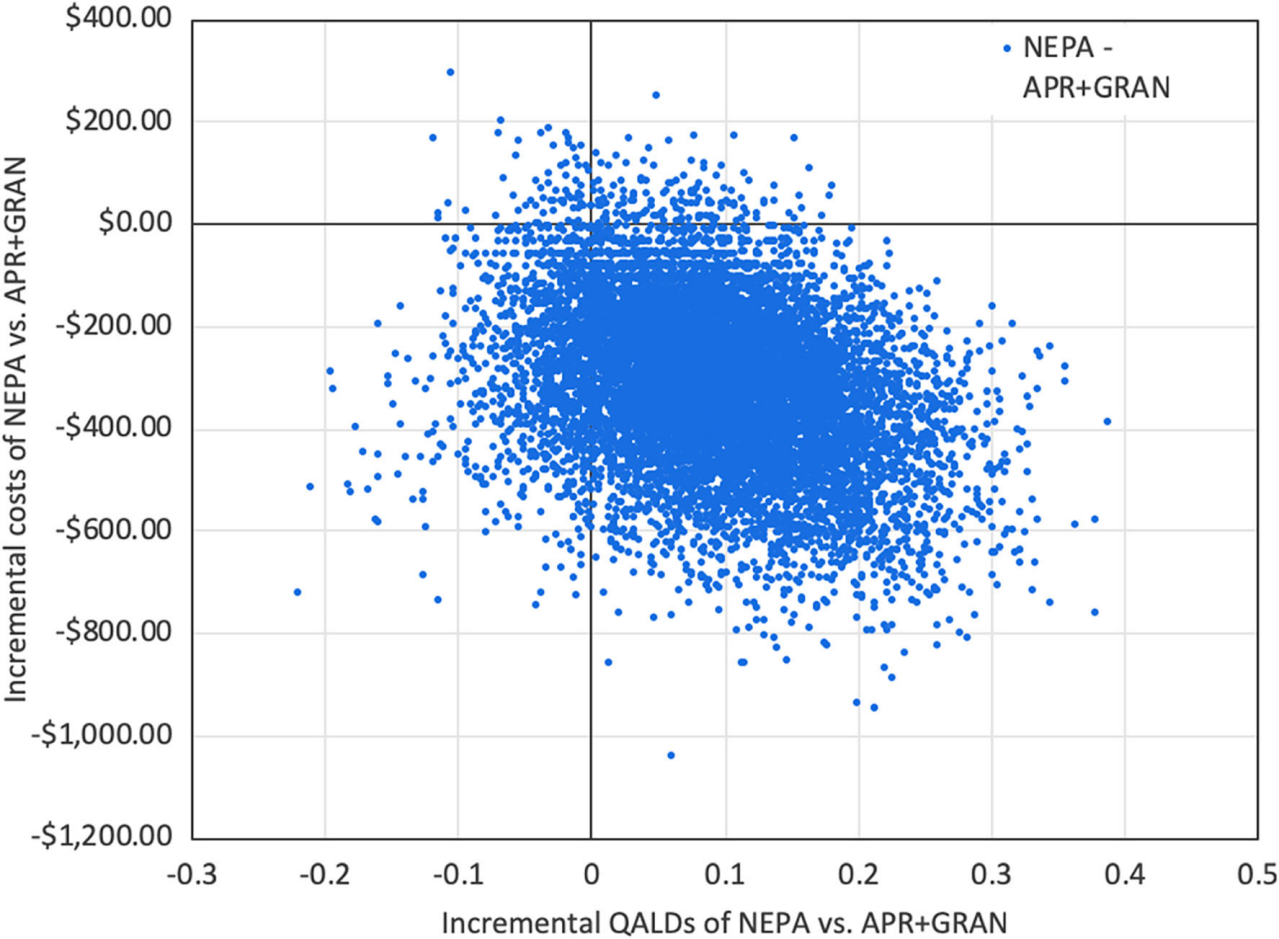
Scatter plot of incremental effects and costs. Costs in 2018 US dollars. *APR*, aprepitant; *GRAN*, granisetron; *NEPA*, netupitant and palonosetron; *QALD*, quality-adjusted life-day

Compared to the use of APR + GRAN, the use of NEPA resulted in numerically better (+1.4 percentage points) CR rates (73.8% vs 72.4%; 95% CI −4.6% to 7.7%), a 2.6-percentage point reduction of severe CINV (i.e., VAS > 80 mm) (4.1% vs 6.7%; 95% CI −5.8% to 0.5%) and a 3.8-percentage point reduction of patients with 3 or more days of emetic episodes or rescue medication use (8.5% vs 12.3%; 95% CI −8.2% to 0.0%). Compared to APR + GRAN, NEPA resulted in a non-significant gain of 0.09 QALDs (95% CI −0.06 to 0.25) and a statistically significant total per-patient cost reduction of $309 (95% CI $4 to $626). The latter was attributable to a mean (95% CI) decrease of $258 (−$46 to $572) in medical costs of CINV-related events ($409 [$215 to $612] vs $668 [$412 to $931]), a $45 reduction in study drug costs ($531 vs $577), and a $5 (−$1 to $15) reduction in rescue medication costs ($3 [$1 to $5] vs $8 [$2 to $17]). The results of the joint bootstrap and PSA simulations are presented as incremental effects and costs in Fig. 1. Importantly, NEPA resulted in lower costs and higher QALDs (and hence was considered “economically dominant”) in 86.5% of joint bootstrap and PSA simulations (i.e., simulations located in the bottom-right quadrant of Fig. 1). The probability of NEPA being cost-effective at a WTP threshold of at least $25,000 was 97.8% and the NMB was $315.

### Sensitivity and subgroup analyses

Table 4 provides detailed results of sensitivity and subgroup analyses. Overall, these confirm the robustness of the base-case results, namely that NEPA is cost-effective. Of note, NEPA was particularly highly cost-effective when the 8.5-h vomiting and/or retching threshold was used to define severe nausea, which resulted in fewer patients having severe nausea in the NEPA treatment group (1.94% vs 6.49%; *P* = 0.001), and in the metastatic population. NEPA remained highly cost-effective (and cost-saving) even when the costs of CINV were assumed to be lower, the ITT population was used (instead of the FAS), and the analysis was restricted to the metastatic population.

**Table 4:** Cost-effectiveness analyses results

	QALDs^a^			Cost^b^			NMB^c^	Probability NEPA is cost-effective at a WTP per QALY of:	
Analysis	NEPA	APR + GRAN	Difference (95% CI)	NEPA	APR + GRAN	Difference (95% CI)		$25,000	$100,000
Base-case	4.04	3.95	0.09 (−0.06 to 0.25)	$943	$1252	$309 ($4 to $626)	$315	97.8%	98.0%
Sensitivity analyses									
− 25% study drug cost	4.04	3.95	0.09 (−0.06 to 0.25)	$943	$1241	$298 (−$8 to $614)	$304	97.4%	97.6%
+ 25% study drug cost	4.04	3.95	0.09 (−0.06 to 0.25)	$943	$1264	$320 ($15 to $637)	$326	98.2%	98.2%
− 25% HCRU cost	4.04	3.95	0.09 (−0.06 to 0.25)	$841	$1085	$245 ($15 to $488)	$251^d^	98.3%^d^	98.5%^d^
+ 25% HCRU cost	4.04	3.95	0.09 (−0.06 to 0.25)	$1046	$1419	$374 (−$8 to $762)	$380^d^	97.4%^d^	97.6%^d^
HCRU cost truncated at 6 days	4.04	3.95	0.09 (−0.06 to 0.25)	$748	$934	$186 ($26 to $357)	$192	98.9%	99.1%
− 50% of 6-day HCRU cost	4.04	3.95	0.09 (−0.06 to 0.25)	$641	$759	$118 ($38 to $217)	$124	99.8%	99.7%
ITT population	4.01	3.94	0.07 (−0.06 to 0.24)	$1028	$1249	$221 (−$80 to $551)	$226	93.2%	93.6%
Threshold for severe nausea									
VAS = 70	4.04	3.95	0.09 (−0.06 to 0.25)	$1112	$1586	$474 ($120 to $857)	$480	99.5%	99.5%
VAS = 90	4.04	3.95	0.09 (−0.06 to 0.25)	$799	$1014	$215 (−$43 to $460)	$221	95.1%	95.7%
> 8.5 h vomiting and/or retching	4.04	3.95	0.09 (−0.06 to 0.25)	$727	$1229	$502 ($248 to $802)	$508	100.0%	100.0%
Subgroups									
Metastatic disease	4.06	3.80	0.26 (−0.07 to 0.46)	$1041	$1690	$649 ($276 to $1026)	$667	100.0%	100.0%
Non-metastatic disease	4.02	4.02	0.00 (−0.13 to 0.13)	$870	$1040	$169 (−$117 to $415)	$169	86.0%	85.5%
Lung cancer	4.13	4.00	0.14 (−0.03 to 0.28)	$857	$1143	$285 ($8 to $573)	$295	98.2%	98.5%
No lung cancer	3.85	3.86	−0.01 (−0.16 to 0.15)	$1115	$1449	$333 (−$45 to $659)	$332	95.2%	94.5%

*APR*, aprepitant; *CI*, confidence interval; *GRAN*, granisetron; *HCRU*, healthcare resource use; *ITT*, intent-to-treat; *NEPA*, netupitant and palonosetron; *NMB*, net monetary benefit; *QALD*, quality-adjusted life-day; *QALY*, quality-adjusted life-year; *VAS*, visual analogue scale; *WTP*, willingness to pay

^a^For reference, the QALDs of 5 days of complete protection (CP) are 4.5

^b^Adjusted to 2018 US dollars

^c^Defined as (QALD difference ÷ 365.25 × $25,000 - cost difference). A positive NMB implies NEPA is cost-effective at the $25,000 per QALY threshold. The higher the NMB, the more cost-effective NEPA is

^d^The NMB is higher (i.e., better for NEPA) when CINV costs are higher, reflecting the fact that NEPA on average prevents CINV. However, the percentage of model simulations in which NEPA is cost-effective appears lower when CINV costs are higher. This reflects the uncertainty/variability in the percentage difference in patients with severe nausea

## Discussion

Limited data exist to directly compare NK1 RA prophylaxis regimens. This study is, to the best of our knowledge, the first to evaluate the economic impact of the selection of an NK1 RA based on actual clinical outcomes from a head-to-head randomized trial comparing two NK1 RA-containing regimens. The consequences of CINV are meaningful from both clinical and economic perspectives, and consideration of opportunities for maximizing outcomes and cost-effectiveness is highly warranted at this time of increasing scrutiny of the cost of cancer treatment.

In our analysis, NEPA showed favorable outcomes against APR + GRAN, with a numerically greater proportion of patients (73.8% vs 72.4%; 95% CI -4.6% to 7.7%) experiencing CR, as well as improvements in severe nausea (VAS > 80 mm) and prolonged CINV (≥ 3 days). Our economic analysis utilized the patient-level data from which those findings were derived to determine that NEPA resulted in statistically significant cost savings and a non-significant increase in QALDs. The significant reduction in per-patient cost resulted from a lower study drug cost and a decrease in CINV-related costs (HCRU and rescue medication cost) due to NEPA’s higher efficacy in preventing severe nausea. A key strength of the methods used in our analysis is the reliance upon the individual patient-level data. The latter allowed us to conduct the economic analyses using a stochastic approach (i.e., full sensitivity analysis) to more fully capture the uncertainty and variability inherent to the data. The sensitivity analyses confirmed the robustness of the results and demonstrated that NEPA is cost-effective at conservative WTP thresholds and various thresholds for defining nausea severe enough to require acute care. NEPA was also cost-effective when severe nausea was defined by a threshold based on patient-reported duration of vomiting and/or retching (> 8.5 h). Moreover, NEPA remained cost-effective in the ITT population despite the four patients in the NEPA study arm assumed to be treatment failures due to not receiving study drug. The analyses demonstrated NEPA’s cost-effectiveness over different time horizons (6 and 10 days) post-HEC and indicated a considerable cost savings potential of NEPA versus APR + GRAN over the course of the complete chemotherapy. In the subgroup of patients with metastatic disease, the cost savings of NEPA versus APR + GRAN were especially high.

Our results are consistent with prior analyses of direct CINV costs in the 5 days post-HEC in the US [31] as well as other analyses evaluating the cost-effectiveness of NEPA versus other NK1 RA- and 5-HT3 RA-containing regimens. Restelli et al. evaluated the incremental cost-utility from the Italian healthcare perspective of NEPA versus APR plus PALO, fosaprepitant (fAPR) plus PALO, APR plus ondansetron (ONDA), and fAPR plus ONDA in patients receiving HEC. Compared to all four comparator regimens, NEPA resulted in decreased incremental medical cost (€30–€71) and increased incremental QALDs (0.08–0.26) in HEC patients [32]. Similarly, from a British healthcare perspective, NEPA was the dominant strategy in HEC patients, resulting in a reduction of costs and a gain of QALDs versus APR + PALO [20]. The results are also consistent with the finding—noted within the National Comprehensive Cancer Network (NCCN) guidelines for antiemesis [8]—that netupitant is effective at decreasing delayed nausea. One possible basis for the relative benefits of NEPA may be that both components of NEPA have half-lives longer than alternative injectable 5-HT3 RA and NK1 RA agents, potentially enhancing CINV prevention in the delayed phase [33].

Both the 5-HT3 RA and the NK1 RA agents differed between arms in the underlying clinical trial. Many clinical studies have evaluated comparative 5-HT3 agents; this is the first to assess comparative NK1 RA agents head-to-head. The advantages shown for NEPA—in terms of fewer overall days of CINV events, shorter overall duration of vomiting, and fewer patients with ≥ 3 days of CINV—all suggest a benefit in the delayed stage, which has been typically attributed to the NK1 RA contribution to prophylaxis.

Although the numerical percentage point difference between NEPA and APR + GRAN in avoided CINV events is not large, the prevalence of chemotherapy use makes these small differences meaningful when considered in the aggregate at a population level. Specifically, when further extrapolating the results of 1 cycle to a full treatment course averaging 4.5 cycles (as the efficacy of NEPA has shown consistency across multiple cycles in other studies [34]), the use of NEPA versus APR + GRAN could result in an average per-patient cost reduction of $1391 per course of therapy. When applied to the total patient population randomized to NEPA in the trial (*n* = 412), these amount to a savings of $572,886. These savings take on added meaning given that CINV recently has been recognized as an opportunity for quality improvement and cost reduction, included in both the definition of what may be the first medical oncology outcome measure (OP-35) imposed by CMS [6] and the landmark finding by Basch et al. showing that monitoring chemotherapy symptoms including nausea and emesis improved survival [3]. The incomplete physician adherence with recognized antiemesis guidelines repeatedly reported in HEC [35, 36] suggests that NEPA’s potential for cost and quality improvement in CINV, based on results from a head-to-head clinical trial, is worthy of consideration.

### Limitations

The analysis had the following limitations. First, the clinical trial did not record CINV-related cost or utilities and these had to be assigned retrospectively based on published values. However, sensitivity analyses indicated that NEPA remained cost-effective when these assumptions were tested. Secondly, the clinical trial was conducted in Asia rather than in the US. However, there is not a basis to expect materially different outcomes within a US population considering the pharmacokinetic profiles of netupitant and palonosetron in Asian and Caucasian patients [37]. Furthermore, the trial tested the use of oral NK1 agents and GRAN rather than the more commonly used ONDA; however, the antiemetic efficacy of these agents is similar [38]. A separate analysis (data not shown) indicates that NEPA would likely have remained cost-saving ($108) versus APR + GRAN if the price of 1-day intravenous APR (150 mg; $335) [23] was used instead of the price of 3-day oral APR. In addition, our analysis does not ascribe any CINV-related HCRU costs for mild to moderate nausea (VAS ≤ 80 mm) and, as a result, might underestimate the total cost per patient. The cost-utility findings reflect the state of patients enrolled in a clinical efficacy trial. As such, these results have high internal validity; however, the degree of external validity might be lower when extrapolating these results to specific patient populations with potentially different antiemetic response rates. Other downstream or indirect costs that could result from CINV, such as those associated with early discontinuation of chemotherapy or missed work among patients and caregivers, were not considered; however, assuming their impact would be associated with the degree of CINV, the results would likely continue to favor NEPA. The present analysis may be conservative as it excluded other benefits of preventing CINV, such as the potential utility gains beyond the initial 5-day post-chemotherapy initiation, the benefits of CINV prevention in subsequent cycles, the reduction in CINV-related chemotherapy discontinuation, or indirect costs.

## Conclusions

Using patient-level outcomes data from a large comparative phase 3 trial as well as conservative cost and utilities assumptions, this analysis suggests that NEPA improves CINV outcomes at lower cost and is cost-effective relative to aprepitant-based regimens in CINV prevention for US patients receiving HEC. The cost reduction is principally due to a decrease in the occurrence of severe nausea with its associated medical cost and, to a lesser extent, a lower drug cost. This result is aligned with cost-effectiveness models assessing NEPA against comparator antiemetic prophylaxis, and supports the use of NEPA within oncology practices seeking to improve adherence to prophylaxis and optimize patient outcomes and cost-effectiveness.

## Electronic supplementary material

ESM 1 (DOCX 19.4 kb)

## Data Availability

All the data used in the analyses are presented in the manuscript. The authors collectively retained control of the data used in the analysis. We agree to allow the journal to review the data if requested, under a specific confidentiality agreement.
