# Supplementary material for: Cost-effectiveness of a fixed combination of netupitant and palonosetron (NEPA) relative to aprepitant plus granisetron (APR + GRAN) for prophylaxis of chemotherapy-induced nausea and vomiting (CINV): a trial-based analysis
Source: Support Care Cancer. 2019 Jun 3;28(2):857–66. doi: 10.1007/s00520-019-04824-y (PMC6954135; doi:10.1007/s00520-019-04824-y)
Supplement: Supplementary file 1 — (DOCX 19.4 kb) [file 520_2019_4824_MOESM1_ESM.docx]

**Supplementary Table 1. Model Input Parameters and Distribution for the Sensitivity Analyses**

| Medication | Dosage | Baseline Cost^a^ | Low  (90% x baseline) | High (110% x baseline) | Distribution for PSA | Source |
| --- | --- | --- | --- | --- | --- | --- |
| **Rescue medication, Oral** |  |  |  |  |  |  |
| Domperidone | 10 mg | $0.73 | $0.65 | $0.80 | Univariate | Red Book 2018 [23] |
| Estazolam | 2 mg | $0.60 | $0.54 | $0.66 | Univariate | Red Book 2018 [23] |
| Famotidine | 20 mg | $0.06 | $0.05 | $0.07 | Univariate | Red Book 2018 [23] |
| Metoclopramide | 10 mg | $0.07 | $0.06 | $0.07 | Univariate | Red Book 2018 [23] |
| Metoclopramide | 5 mg | $0.06 | $0.06 | $0.07 | Univariate | Red Book 2018 [23] |
| Olanzapine | 5 mg | $0.09 | $0.08 | $0.09 | Univariate | Red Book 2018 [23] |
| Omeprazole | 20 mg | $0.06 | $0.05 | $0.07 | Univariate | Red Book 2018 [23] |
| **Rescue medication, IV/IM** |  |  |  |  |  |  |
| Dexamethasone intensol | 4 mg | $0.47 | $0.42 | $0.51 | Univariate | CMS 2018a [22] |
| Dexamethasone intensol | 5 mg | $0.59 | $0.53 | $0.64 | Univariate | CMS 2018a [22] |
| Dexamethasone intensol | 8 mg | $0.94 | $0.84 | $1.03 | Univariate | CMS 2018a [22] |
| Granisetron | 3 mg | $9.51 | $8.56 | $10.46 | Univariate | CMS 2018a [22] |
| Metoclopramide hydrochloride | 10 mg | $0.93 | $0.83 | $1.02 | Univariate | CMS 2018a [22] |
| Metoclopramide hydrochloride | 20 mg | $1.85 | $1.67 | $2.04 | Univariate | CMS 2018a [22] |
| Esomeprazole | 40 mg | $8.00 | $7.20 | $8.80 | Univariate | Red Book 2018 [23] |
| Esomeprazole | 80 mg | $16.00 | $14.40 | $17.60 | Univariate | Red Book 2018 [23] |
| Ondansetron hydrochloride | 8 mg | $0.67 | $0.60 | $0.74 | Univariate | CMS 2018a [22] |
| Palonosetron hydrochloride | 0.25 mg | $203.02 | $182.72 | $223.32 | Univariate | CMS 2018a [22] |
| Ondansetron hydrochloride | 5 mg | $0.42 | $0.38 | $0.46 | Univariate | CMS 2018a [22] |
| Dexamethasone sodium phosphate | 5 mg | $0.59 | $0.53 | $0.64 | Univariate | CMS 2018a [22] |
| Diphenhydramine hydrochloride | 1 mg | $1.18 | $1.07 | $1.30 | Univariate | CMS 2018a [22] |
| Promethazine hydrochloride | 25 mg | $1.23 | $1.11 | $1.36 | Univariate | CMS 2018a [22] |

Abbreviations: CMS, Centers for Medicare & Medicaid Services; IM, intramuscular; IV, intravenous.

^a^ *Costs in 2018 US dollars*.
